# Negative catheter angiography after vascular contrast extravasations on computed tomography in blunt torso trauma: an experience review of a clinical dilemma

**DOI:** 10.1186/1757-7241-20-46

**Published:** 2012-07-07

**Authors:** Kuo-Ching Yuan, Yon-Cheong Wong, Being-Chung Lin, Shih-Ching Kang, Erh-Hao Liu, Yu-Pao Hsu

**Affiliations:** 1 Trauma and Critical Care Center, Division of General Surgery, Department of Surgery, Chang-Gung Memorial Hospital, Chang-Gung University, Linkou, Taiwan; 2 Division of Emergency and Critical Care Radiology, Department of Medical Imaging and Intervention, Chang-Gung Memorial Hospital, Chang-Gung University, Linkou, Taiwan

**Keywords:** Trauma, Blunt abdominal injury, Pelvic injury, Contrast extravasation, Computed tomography, Angiography, Embolization, Negative angiography

## Abstract

**Background:**

Catheter angiography is often arranged when vascular contrast extravasations on computed tomography (VCEC) presents after blunt torso trauma. However, catheter angiograph can be negative for bleeding and further management about this condition is not well discussed. The purpose of this study was a review of our experience of this discrepancy and to propose management principle.

**Methods:**

We conducted a retrospective analysis of patients who received catheter angiography due to VCEC after blunt torso trauma at a level one trauma center in Taiwan from January 1, 2006 to December 31, 2009. Patient data abstracted included demographic data, injury mechanism, Injury Severity Score, vital signs and laboratory data obtained in the emergency department, CT and angiography results, embolization status, rebleeding and outcome. Analysis was performed according to angiographic results, VCEC sites, and embolization status.

**Results:**

During the study period, 182 patients received catheter angiography due to VCEC, and 48 (26.4%) patients had negative angiography. The kidney had the highest incidence (31.7%) for a discrepant result. Non-selective proximal embolization under negative angiography was performed mostly in pelvic fracture and spleen injury. Successful treatment without embolization after negative angiography was seen in the liver, kidney and pelvic fractures. However, some rebleeding happened in pelvic fractures with VCEC even after embolization on negative angiography.

**Conclusions:**

A negative catheter angiography after VCEC is possible in blunt torso trauma, and this occurs most in kidney. Embolization or not under this discrepancy requires an integrated consideration of injury site, clinical presentations, and the risk of rebleeding. Liver and kidney in blunt torso trauma can be managed successfully without embolization when catheter angiography is negative for bleeding after VCEC.

## Background

Computed tomography (CT) scan with intravenous (IV) contrast is currently widely used in evaluation of trauma patients [1-3]. Vascular contrast extravasations on CT (VCEC), which indicates the leaking of contrast medium from vessels, appearing as a localized or diffuse high density region on the CT scan, is regarded as an evidence of active bleeding or vascular injury. Further interventions, such as surgery or transcatheter artery embolization (TAE), are usually indicated for hemostasis if VCEC is present with other unstable presentations (persistent shock, poor response to fluid resuscitation) [2,4]. As a result of advances in interventional radiology, TAE is now more often the first choice in blunt solid organ trauma when the patient is hemodynamically stable [2,4-6], or has a pelvic fracture with shock and arterial bleeding [7].

Discrepancies are sometimes encountered between the CT scan and the following catheter angiography. There may be no definite bleeding or vascular lesions identified by catheter angiography despite the presence of VCEC. Selective embolization is difficult to perform in these cases because CT scan itself does not always provide a precise location for embolization. None-selective embolization, however, can result in unnecessary tissue ischemia and other adverse effects, which makes the multiple trauma patient more complicated. If embolization is not performed, there is a higher risk of failed non-operative treatment when VCEC is present [4,8]. Surgery is usually not indicated for those blunt torso trauma patients who do not exhibiting other surgical indications.

Herein, we review our experience of this situation at a single, level-1 trauma center in Taiwan. With the evaluation of association between injuries and the discrepant results, the purpose of this study is to elucidate some management suggestions for this discrepancy after blunt torso trauma.

## Methods

This was a retrospective chart review study and permission from the Institutional Review Board was obtained. The medical records of all patients who were admitted into the Department of Trauma and Emergency Surgery for blunt torso trauma from January 1, 2006 to December 31, 2009 were reviewed from our trauma registry system. The patients who received catheter angiography after blunt torso trauma were selected first. Those who did not have a torso CT scan before angiography were excluded first. Patients who had torso CT scan before angiography but did not have VCEC or received angiography under indications other than VCEC were also excluded. Patients who had clear record that VCEC as the indication for angiography on angiography report or other medical documents (such as surgical consult sheet) were selected in this study.

**Figure 1:**
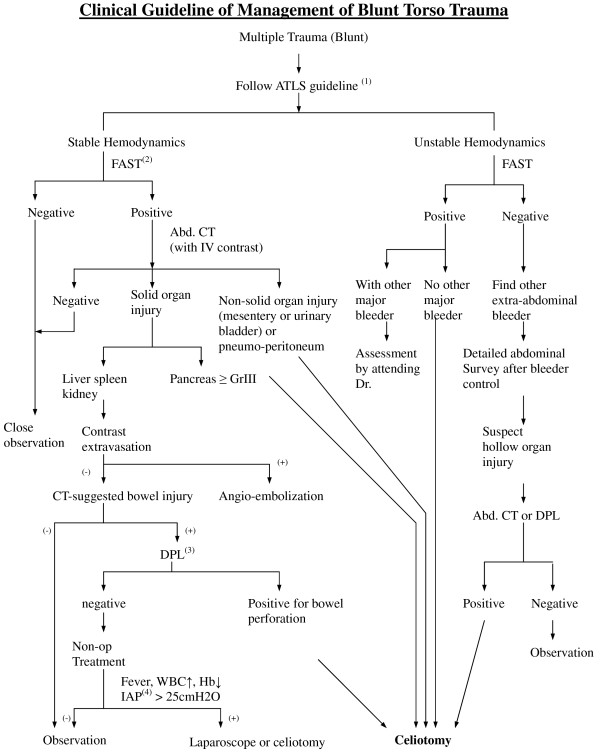
**Protocol for management of blunt torso trauma **.(1). ATLS guideline: Primary survey (ABC), Resuscitation: challenge Lactated Ringer 2L if SBP < 90 mmHg, Secondary survey, Routine laboratory tests, Trauma Series image (C-spine lateral view, CXR, Pelvis A-P). (2). FAST: Focused Abdominal Sonography of Trauma. (3). DPL: Diagnostic Peritoneal Lavage. (4). IAP: Intra-Abdominal Pressure.

In our hospital, blunt torso trauma patients undergo initial management following our protocol based on the Advanced Trauma Life Support (ATLS) guidelines (Figure 1). Patients who are in stable hemodynamic status or have good response to fluid resuscitation will receive a CT scan with IV contrast if abdomen or pelvic injuries are suspected. CT scan is performed using a 16-multidetector CT machine (LightSpeed QX/i Scanner, General Electric Medical Systems, Milwaukee, WI, USA), which is located adjacent to the surgical resuscitation room. IV contrast agents are routinely administrated unless contraindicated. A uniphasic injection of 100–120 ml of contrast agent is given to the patient at a rate of 1–3 ml/s, and images of 5–10 mm collimation and 5–8 mm reconstruction intervals are obtained 60–70 s after the start of intravenous contrast medium administration. If CT detects vascular contrast extravasations in solid organs or pelvic fracture site and the hemodynamic status is relatively stable (Systolic blood pressure≧90 mmHg with resuscitation in progress), catheter angiography is indicated. Other indications for catheter angiography include unstable pelvic fracture with persistent shock; persistent hypotension despite aggressive resuscitation (cavitary bleeding excluded); and difficult hemorrhage control during operation, for which catheter angiography can be used as an adjunct to surgery.

Catheter angiography begins with a right or left femoral artery puncture using the Seldinger technique. A 5-Fr introducer sheath is then applied, followed by a 5-Fr pigtail catheter (Cordis, Miami, FL, USA). An abdominal aortography is obtained first for anatomical evaluation and the location of possible bleeding. Then, a selective angiography study is performed according to the bleeding sites unveiled on the CT scan or abdominal aortography. For the selective catheterization, a diagnostic catheter is inserted into the target vessel followed by another contrast injection for precise localization and evaluation. Either contrast extravasation or vascular pseudoaneurysm is regarded as positive for bleeding.

Patients with positive catheter angiography always undergo embolization according to our protocol, unless specific issues prevent embolization. Embolization is performed using metallic coils/microcoils (Cook^®^, Bloomington, IN, USA) or gelfoam (Curaspon®, CuraMedical BV, Netherlands), depending on the operating radiologist. However, there was no established protocol for addressing a negative catheter angiography in our hospital during study period; further management for these angiography negative patients was at the discretion of the on duty trauma surgeon and radiologist. Some patients received none-selective embolization, and some received only conservative treatment with blood transfusions. It was a decision according to the surgeon’s personal experience and the patient’s clinical condition at that time.

All official reports of CT scan and catheter angiography in our hospital were issued by board-certified radiologist with specializations in trauma. So the official reports were used as the final interpretation of the images without further reevaluation. There were some patients who were referred from another hospital at which a CT scan was performed. They were marked as “referred patients” and there was no official CT report for them. The referred patient’s medical record or the referring note was used as the final interpretation of their CT scan. A diagnosis of VCEC was confirmed from a clear description on the official CT report or on the medical records of the referred patients.

All these patients, whether they received embolization or not, were admitted to Trauma Intensive Care Unit (TICU) after first angiography for close monitoring with arterial access sheath left on the femoral artery. If rebleeding was highly suspected from clinical presentations such as hypotension, tachycardia, low urine output, persistent metabolic acidosis, or unstable hemoglobin requiring frequent transfusion; the patient would receive a repeat angiography; or surgery if clinical condition was not suitable for angiography. A rebleeding happened within 72 h after first angiography is considered failure of previous management. An analysis about rebleeding is also performed.

Data collected included patient demographic data, injury mechanism, Injury Severity Score (ISS), initial vital signs in triage, laboratory data obtained in the emergency department (ED), where the CT scan was performed (referred patient or not), vascular extravasation sites on CT scan, catheter angiography result, embolization status, rebleeding, and patient outcome. If a patient had more than one site of vascular extravasation detected on CT scan but not all bleeders detected on catheter angiography, the patient was assigned to the angiography negative group for demographic data analysis. But the bleeding sites were analyzed separately.

All numerical data were expressed as the mean ± standard deviation(SD). SPSS software, version 16 (SPSS, Chicago, IL, USA) was used for all analyses. A *p* value of 0.05 was regarded as statistically significant. Continuous numerical variables were analyzed by a two-sample *t*-test or one-way analysis of variance (one-way ANOVA). Categorical variables were analyzed by the *χ*^2^ test or Fischer’s exact test.

## Results

**Figure 2:**
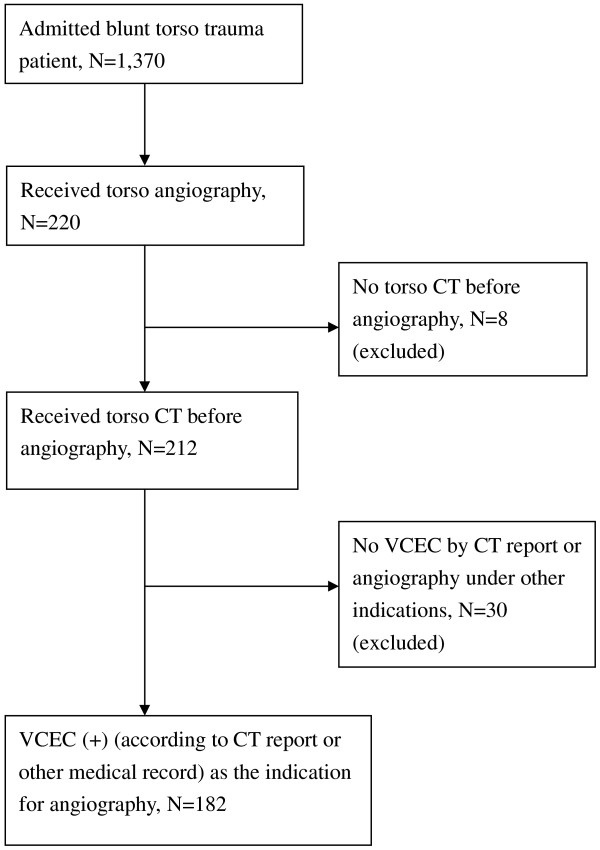
Flow diagram for patient selection.

There were 1,370 patients admitted to the Department of Trauma and Emergent Surgery due to blunt torso trauma in our trauma registry database during the 4-year study period, and 220 (16.1%) patients received catheter angiography. Eight patients were excluded because they did not have a CT scan before catheter angiography. Thirty patients did not have VCEC and received catheter angiography under other indications, and they were also excluded. Thus, 182 patients who received catheter angiography due to VCEC were included in our study (Figure 2).

There were 54 females and 128 males, and the mean age was 33.8 ± 16.2 years. Most patients were severely injured, and the mean Injury Severity Score was 25.2 ± 10. Sixty-six (36.3%) patients were referred patient. There were 16 patients had more than one site of VCEC. Based on catheter angiography result, 134 (73.6%) patients were placed in the angiography positive (AP) group, and 48 (26.4%) patients were placed in the angiography negative (AN) group (Table 1). An analysis to compare the AP and AN was performed for demographic data, vital signs in triage, injury mechanism, Injury Severity Score, initial laboratory results obtained in the ED, and referral status. None of these factors was statistical significant between the AP and AN groups (Table 1).

**Table 1:** General data between angiography positive (AP) and angiography negative (AN) patients

	**AP**	**AN**	***p***
Patient no.	134 (73.6%)	48 (26.4%)	
Age(yr)	33.9 ± 16.5	33.5 ± 15.4	0.87
Sex (M/F)	98/36	30/18	0.17
ISS	25.6 ± 10.2	24.0 ± 9.4	0.33
Pulse (beat/minute)	105.4 ± 25	100.2 ± 28.6	0.27
SBP (mmHg)	107.7 ± 34.5	103.2 ± 38	0.47
Hemoglobin (g/dL)	11.3 ± 2.7	11.5 ± 2.4	0.57
Platelet (1000/uL)	180.1 ± 72.4	189 ± 73.2	0.47
Trauma mechanism			0.11
Motor bike accident	92	27	
Motor vehicle accident	15	7	
Falling	14	10	
Fight	2	3	
Hit by objects	2	0	
Pedestrian accident	9	1	
Outcome			0.54
survived	124	43	
died	10(8.1%)	5(11.6%)	
Referred patient	49 (57.7%)	17 (54.8%)	0.89

ISS: Injury Severity Score; SBP: Systolic Blood Pressure; DBP: Diastolic Blood Pressure.

Further analysis was performed according to the sites of VCEC. Within the 182 included patients, there were 198 vascular contrast extravasations distributed in five different injury sites (liver, spleen, kidney, pelvic, retroperitonium). In the following angiography, there were only 149 bleeding detected, and 49 (24.8%) sites were negative for bleeding (Table 2). For the 149 angiography positive sites, embolization was performed on 147 sites. One spleen injury did not receive embolization because technical difficulty caused by a congenital variation of vessels. Embolization was also abandoned in one kidney injury related bleeding due to technique problems. They both received emergent operation immediately after angiography.

**Table 2:** Result of CT, angiography and embolization of all patients

**Injury site**	**Liver**	**Spleen**	**Pelvic**	**Kidney**	**Retroperitonium**	**Total**
NO. of CT extravasations	50 (25%)	47 (24%)	51 (26%)	41 (21%)	9 (5%)	198
Angiography						
positive	38	38	37	28	8	149 (75.3%)
negative	12 (24%)	9 (19.2%)	14 (27.5%)	13 (31.7%)	1 (11.1%)	49 (24.7%)
Embolization						
Angio-positive with embolization	38	37	37	27	8	147
Angio-positive, no embolization	0	1	0	1	0	2
Angio-negative with embolization	1 (8.3%)	7 (77.8%)	12 (85.7%)	1 (7.7%)	0	21 (42.9%)
Angio-negative, no embolization	11 (91.7%)	2 (22.2%)	2 (14.3%)	12 (92.3%)	1	28 (57.1%)

A negative catheter angiography after VCEC occurred most often in kidney and the incidence was 31.7% (Table 2); whereas the retroperitoneum had the lowest incidence (11.1%). For the 49 catheter angiography negative sites, embolization was done in 21 (42.9%) sites (Table 2). Embolization under a negative angiography was performed at the proximal site main supplying artery (right hepatic/left hepatic artery, splenic artery, upper or lower branch of renal artery, right/left internal iliac artery). 85.7% of pelvic fracture related bleeding and 77.8% of spleen injuries were embolized under a negative catheter angiography. However, if the injury site was liver or kidney, there was a trend toward conservative treatment. The none-embolization rate was 91.7% in liver injury and 92.3% in kidney injury if the catheter angiography was negative (Table 2).

There were 18 patients who had rebleeding requiring subsequent procedure within 72 h after first angiography. Thirteen of them were in the AP group and all received embolization in the first angiography (liver = 3, spleen = 3, pelvic fracture = 1, kidney = 5, retroperitonium = 1). Five patients were in the AN group, including two pelvic fracture patients who were angiography negative but both had embolization in the first angiography. Other three rebleeding AN patients (liver = 1, spleen = 1, and kidney = 1) did not receive embolization in the first angiography. With respect to different injury site, we found that liver, kidney and pelvic could be managed successfully without embolization when angiography was negative (Table 3).

**Table 3:** Treatment results of all angiography negative (AN) patients, with or without embolization

	**Rebleeding**	**Success treatment (%)**
Liver (*n* = 12)		
E = 1	0	1
NE = 11	1	10 (90.9%)
Spleen (*n* = 9)		
E = 7	0	7
NE = 2	1	1 (50%)
Pelvic (*n* = 14)		
E = 12	2	10
NE = 2	0	2 (100%)
Kidney (*n* = 13)		
E = 1	0	1
NE = 12	1	11 (91.7%)

*n*: AN patient number.

E: Angiography negative with embolization.

NE: Angiography negative without embolization.

After treatment, 15 (8.3%) patients died. Ten of these patients were in the AP group, and all received embolization. Six of the ten AP patients died of shock related organ damage, which leaded to multiple organ failure after admission. Two had severe brain injury, and the other two developed sepsis with multiple organ failure during admission. Five patients in the AN group died. One had liver injury without embolization and died of pneumonia with sepsis. The other four patients had pelvic fracture- related bleeding, and all received embolization after negative catheter angiography. Two of them died of shock related organ damages; one died of pneumonia with sepsis; and one died of severe brain injury.

## Discussion

As early as 1989, VCEC was first described as an indicator of bleeding in a spleen injury patient [4]. Nowadays, VCEC not only is considered a strong evidence of bleeding, but also indication for surgery or angioembolization [9-11]. TAE is usually used more than surgery after VCEC in blunt abdominal solid organ injury [9,12,13], pelvic fracture related bleeding [7,14], and retroperitoneal bleeding [15]. The reported success rate of TAE in blunt torso trauma can be as high as 90–100% [5,16,17]. However, there is not much discussion regarding a negative catheter angiography after VCEC. A discrepant result between CT scan and the following angiography is very possible because CT scan only has76% sensitivity and 80% positive predictive value for detecting bleeding or vascular injury [18]. In our study, the incidence of discrepant results was 26.4% in blunt torso trauma. In a brief report regarding 30 pelvic fracture patients, the reported rate of discrepancy was approximately 11.1% [14]. There are some possible causes for this discrepancy. First, it is possible that some VCEC were actually venous bleedings or non-vascular contrast leakage. In that case, the following catheter angiography for artery bleeding would be negative. The second possible cause is that small fragmented parenchyma of solid organs (liver, spleen, kidney) could have been misinterpreted as vascular contrast extravasation on CT scan; so no bleeding was observed in the following angiography. The third possibility is spontaneous endogenous hemostasis. The endogenous hemostasis mechanism could have stopped the bleeding before catheter angiography.

After analysis, we found that factors such as demographic data, trauma mechanism, ISS, vital signs in triage, ED laboratory results and referral status; were not significant for different catheter angiography results (Table 1). Therefore, none of these factors can be predictive of angiography result after VCEC. The referral status was once a concern. The difference in CT scan protocols between hospitals was presumed to cause bias in the interpretation of CT, and then leaded to more discrepant angiography results. However, the referral status turned out not to be significant in our study. Then we analyzed the angiography results by different sites and kidney is noted to have the highest incidence (31.7%) of negative catheter angiography after VCEC (Table 2). In a further analysis of some severity factors of all VCEC patients, we found that kidney injury patients had better systolic blood pressure, and received least blood transfusion (Table 4). It is possible that because kidney injury patients had better hemodynamic status and more preserved coagulation ability; and their bleeding was therefore more likely to stop spontaneously before angiography. Besides, a contrast leakage from urinary collecting system or misinterpretation of renal parenchyma into VCEC that lead to a negative angiography is also possible in kidney injury.

**Table 4:** Comparison between different injury sites for all VCEC patients and AN patients

**Injury site**	**Liver**	**Spleen**	**Pelvic**	**Kidney**	**Retroperitonium**	***p***
Case no.	(50)/(12)	(47)/(9)	(51)/(14)	(41)/(13)	(9)/(1)	
Age	(29.4 ± 12.6)/(34.1 ± 13.9)	(35.2 ± 15.9)/(31.1 ± 12.4)	(37.6 ± 18.1)/(37.6 ± 19.1)	(30.5 ± 15.2)/(28.7 ± 12.2)	(40 ± 17.4)/(-)	(0.03)/(0.55)
ISS	(22.2 ± 9.6)/(21.8 ± 8.8)	(24.1 ± 9.6)/(21.1 ± 8.1)	(29.7 ± 9.4)/(28.8 ± 10.1)	(25 ± 10.3)/(22.9 ± 8.9)	25.2 ± 11.3/(-)	(0.00)/(0.14)
SBP(mmHg)	(110.2 ± 38.5)/(105.3 ± 43.5)	(111.5 ± 27.9)/(111.7 ± 23.6)	(94.9 ± 39.2)/(83.4 ± 36.8)	(114.3 ± 33.7)/(111.5 ± 47.8)	91.8 ± 22.2/(-)	(0.03)/(0.24)
TBT (unit)	(7.3 ± 7.8)/(8.9 ± 12.9)	(7 ± 5.7)/(3.8 ± 3.7)	(13.6 ± 11.5)/(15.3 ± 10)	(6.6 ± 7.6)/(2.2 ± 2.3)	(8.2 ± 5.6)/(-)	(0.00)/(0.005)

All data were presented in form as (all VCEC patients)/(AN patients).

There is only one patient who was AN in retroperitonium so we did not perform calculation.

ISS: Injury Severity Score.

SBP: Systolic Blood Pressure at Emergency Department.

TBT: Total blood transfusion (including Whole Blood, Packed Red Blood Cell concentrate, Fresh Frozen Plasma) before admitted to TICU.

For all AN patients in our study, embolization was performed most for pelvic fractures (85.7%, Table 2) and spleen injury (77.8%), but less performed for kidney (7.7%) and liver injuries (8.3%). After analysis the demographic and laboratory factors of all AN patients, we noticed that the kidney and liver injury AN patients had relative better blood pressure, and less blood transfusion amount (Table 4). These findings indicate that kidney and liver injury AN patients had better clinical conditions. Therefore, embolization was often aborted in these patients if angiography was negative. On the contrary, up to 85.7% pelvic fracture AN patients in our study were embolized. These patients had the highest ISS, hypothermia, the lowest systolic blood pressure and received most blood transfusion before admitted to TICU (Table 4). In fact, pelvic fracture is regarded as a dangerous, high-energy injury, and embolization is strongly suggested in literatures for unstable condition [19,20]. Moreover, none-selective proximal embolization of the internal iliac artery at the injury site is a preferred procedure in pelvic fracture related bleeding; since superselective TAE is reported to associate with increased risk of rebleeding [7]. Although we had two successful cases using none-embolization in pelvic fracture with VCEC, this decision should be made cautiously.

With respect to spleen injury, more than 70% of spleen injury patients who were angiography negative after VCEC received embolization in our study (Table 2). Although these patients had fair clinical condition (Table 4), most trauma surgeons in our hospital still chose embolization despite of negative angiography. This can be attributed to that VCEC in spleen injury is already considered a strong and dangerous evidence of bleeding, and one negative angiography is not sufficient to totally overrule the significance of VCEC in spleen injury. In fact, there are studies that emphasize the importance of embolization in spleen injury. Dr. Shanmuganathan et al. concludes that almost all spleen-injured patients require intervention if VCEC presents [8]. Additionally, proximal splenic artery embolization with coils, which is used to decrease blood flow, has been considered better than selective distal embolization because distal embolization infarcts more tissue [21]. Therefore, it is recognized as efficient and safe to employ the procedure even when bleeding is not seen on the catheter angiography [22].

In fact, the angiography result is only one of the many considerations in management of complex multiple trauma patients. To embolize or not is usually made with the integration of injury site, clinical presentation and risk of rebleeding for a given site. Liver and kidney injury patients were less embolized in our study and it is possibly due to relative stable clinical condition in comparison to other injury sites. Embolization was done more in pelvic fracture and spleen injury because of the unstable conditions in pelvic fracture and the high bleeding risk in spleen injury. In an analysis of treatment results of all AN patients, none-embolization after negative catheter angiography presents successful cases in liver, pelvic, kidney (Table 3). However, we also noticed rebleeding happened in two AN pelvic fracture patients who had received embolization in first angiography. One of the two spleen injury patients who had VCEC and negative angiography without embolization also had rebleeding. Therefore, aggressive embolization should be done in pelvic fracture and spleen injury with VCEC but negative angiography. Liver and kidney injury with VCEC can be managed more safely without embolization if catheter angiography is negative.

This study has several shortcomings. It is a retrospective and single center study with all of its inherent limitations. The patient number is relative small for statistical analysis so a solid recommendation by statistic result is difficult to make. There was no adjustment for potential confounders (age, pre-existing comorbidities, potential confounding medications, amounts of fluid administered for resuscitation). There are also some factors possibly relevant to our study, which were not been discussed here. For example: the type of CT machine, and the time interval between CT to angiography. As much as 36.3% of the included patients were referred from different hospitals, these factors were sometimes not recorded completely and precise information was difficult to collect. In the future, a study using dynamic CT may be able to overcome the factors causing misinterpretation of the CT (venous bleeding, nonvascular contrast leakage, or fragmented parenchyma) and reduce incidence of negative catheter angiography.

## Conclusion

About 26.4% of blunt torso trauma patients with VCEC will have a negative catheter angiography, and this occurs most often in kidney. Embolization or not under a negative catheter angiography requires an integrated consideration of injury site, clinical presentations, and the risk of rebleeding. Aggressive embolization despite of the negative angiography should be seriously considered in spleen injury and pelvic fracture because of unstable condition and high risk of rebleeding. Liver and kidney injury with VCEC, however, can be managed safely without embolization if angiography was negative.

## Abbreviations

VCEC, Vascular contrast extravasation on computed tomography; CT, Computed tomography; IV, Intravenous; TAE, Transcatheter artery embolization; TICU, Trauma Intensive Care Unit; ISS, Injury Severity Score; AP, Angiography positive; AN, Angiography negative; ED, Emergency department.

## Competing interest

The authors have no competing interests.

## Author’s contributions

KCY performed data analysis and drafted the manuscript. YCW performed image related data collection, analysis and critical revisions to the manuscript. BCL, EHL and SCK performed clinical data collection and critical revisions to the manuscript. YPH conceived the research and complete revisions to the manuscript. Each author has read and approved the final manuscript.
